# Intracellular Organization by Jumbo Bacteriophages

**DOI:** 10.1128/JB.00362-20

**Published:** 2020-12-18

**Authors:** Jingwen Guan, Joseph Bondy-Denomy

**Affiliations:** a Department of Microbiology and Immunology, University of California, San Francisco, San Francisco, California, USA; b Quantitative Biosciences Institute, University of California, San Francisco, San Francisco, California, USA; c Innovative Genomics Institute, Berkeley, California, USA; McGovern Medical School

**Keywords:** jumbo bacteriophage, PhuZ tubulin filament, shell, nucleus-like structure, compartmentalization, protection, CRISPR-Cas immune systems, phage therapy

## Abstract

Since their discovery more than 100 years ago, the viruses that infect bacteria (bacteriophages) have been widely studied as model systems. Largely overlooked, however, have been “jumbo phages,” with genome sizes ranging from 200 to 500 kbp. Jumbo phages generally have large virions with complex structures and a broad host spectrum. While the majority of jumbo phage genes are poorly functionally characterized, recent work has discovered many unique biological features, including a conserved tubulin homolog that coordinates a proteinaceous nucleus-like compartment that houses and segregates phage DNA. The tubulin spindle displays dynamic instability and centers the phage nucleus within the bacterial host during phage infection for optimal reproduction. The shell provides robust physical protection for the enclosed phage genomes against attack from DNA-targeting bacterial immune systems, thereby endowing jumbo phages with broad resistance. In this review, we focus on the current knowledge of the cytoskeletal elements and the specialized nuclear compartment derived from jumbo phages, and we highlight their importance in facilitating spatial and temporal organization over the viral life cycle. Additionally, we discuss the evolutionary relationships between jumbo phages and eukaryotic viruses, as well as the therapeutic potential and drawbacks of jumbo phages as antimicrobial agents in phage therapy.

## Abstract (precis)

Since their discovery more than 100 years ago, the viruses that infect bacteria (bacteriophages) have been widely studied as model systems. Largely overlooked, however, have been “jumbo phages,” with genome sizes ranging from 200 to 500 kbp. Jumbo phages generally have large virions with complex structures and a broad host spectrum. While the majority of jumbo phage genes are poorly functionally characterized, recent work has discovered many unique biological features, including a conserved tubulin homolog that coordinates a proteinaceous nucleus-like compartment that houses and segregates phage DNA.

## INTRODUCTION

Bacteriophages, also known as phages, are viruses that infect bacteria. They are the most abundant biological entities, with an estimated population of 10^31^ virions in the biosphere (1–3). Phages were identified independently as microbes “antagonistic” to bacteria by F. W. Twort and Félix d’Hérelle in 1915 and 1917, respectively (4). Phages exhibit substantial diversity in viral morphology, genome sequence, life cycle, interaction with their hosts, and the distribution pattern in ecosystems (5, 6). The majority (>96%) of phages examined by electron microscopy (EM) are tailed and contain double-stranded DNA (dsDNA) packaged in their capsids, belonging to the order *Caudovirales* (7, 8). These characterized tailed dsDNA phages exhibit extensive variation in genome size, ranging from ∼10 kbp to >500 kbp (6, 9). Large phages with 200- to 500-kbp genomes have been defined as “jumbo phage,” a term introduced by Roger W. Hendrix in 2009 (10, 11). In 2019, a new group of 15 phages with genome sizes over 540 kbp, all of which infect bacteria of the genus *Prevotella*, were sequenced from human and animal gut microbiomes (12). These phages were termed “megaphages” so as to be distinguished from jumbo phages. Most recently, four phages with manually curated and circularized genomes of 634, 636, 642, and 735 kbp have been reported and referred to as “mahaphages” along with the aforementioned megaphages (13). However, culture-based isolation has not yet been reported for mega/mahaphages, and thus, jumbo phages currently represent the most experimentally tractable of the large phages and will be the focus of this review.

To date, more than 150 jumbo phages have been deposited in the GenBank database. The majority of them infect Gram-negative bacterial strains, including *Escherichia*, *Pseudomonas*, *Aeromonas*, *Caulobacter*, *Erwinia*, *Vibrio*, and *Salmonella* strains (14). Only 11 jumbo phages infect Gram-positive bacteria, mostly the *Bacillus* genus (15). Most sequenced jumbo phages belong to the *Myoviridae* family, members of which have long contractile tails, while the rest belong to the *Siphoviridae* family, which have noncontractile tails. Although jumbo phages have frequently been found in a multitude of environments, they may have been overlooked in standard sampling efforts due to their large virions, which cause limited diffusion and tiny plaques on semisolid media (10, 16).

In addition to the core genes encoding viral structural components and genome replication proteins, jumbo phage genomes encode numerous hypothetical proteins that do not match the current sequence databases. For example, the genome sequence of Pseudomonas aeruginosa phage ФKZ, the first jumbo phage sequenced, was initially reported to comprise 306 open reading frames (ORFs), of which only 59 exhibited similarity to proteins with known or predicted functions from diverse organisms (17, 18) (the number of coding sequences was later expanded to 369 based on analysis of the ФKZ transcriptional map [19]). The genome sequences of many jumbo phages have few homologs with previously annotated phages or other microorganisms and are even highly divergent from each other (e.g., ФKZ and PA5oct, both infecting P. aeruginosa) (14, 15, 17, 20–22).

Jumbo phages have genomes sufficiently large to enable them to encode proteins involved in their own replication, transcription, and translation, such as DNA polymerase and RNA polymerase (RNAP) (10, 14, 20, 23–25). RNAPs encoded by jumbo phages are generally divided into two multisubunit RNAP complexes, virion RNAP (vRNAP) and nonvirion RNAP (nvRNAP) (25–27). For example, *Pseudomonas* jumbo phage ФKZ encodes proteins that constitute a vRNAP that is homologous to bacterial RNA polymerase β or β′ subunits. Upon infection, the vRNAP is coinjected with the ФKZ genome into the host and is responsible for the transcription of early phage genes. The nvRNAP of ФKZ appears later during the infection cycle and transcribes middle and late phage genes (see Fig. 3). The concerted action of these two complexes makes ФKZ transcription independent of the host transcription apparatus (19, 27, 28). Some jumbo phages are enriched in translation-related genes, including tRNAs and aminoacyl-tRNA synthetases (17, 29–31). The Xanthomonas citri jumbo phage XacN1, for instance, harbors 56 tRNAs, corresponding to all 20 amino acids, in its genome (30). Although some small phages, such as the cluster M mycobacteriophages, with an average genome size of ∼82 kbp, are known to encode large sets of tRNA genes spanning nearly the entire genetic code, XacN1 represents the largest number of tRNA genes ever reported in any viruses (32). As a consequence of the abundant transcription- and translation-associated genes, the postinfection development of jumbo phages exhibits a high level of independence from the host molecular machinery (19, 26). This independence appears to endow jumbo phages with the versatility of expanded host ranges, such as the above-mentioned jumbo phage XacN1, which has a wider host range than smaller X. citri phages (30).

As large and structurally complex virions, jumbo phages contain more structural proteins than smaller phages. Structural and proteomic analyses revealed that *Pseudomonas* jumbo phage ФKZ, EL, and 201Ф2-1 virions harbor 62, 64, and 89 structural proteins, respectively (33, 34). The relatively large Escherichia coli phage T4, the paradigm for study of the *Myoviridae* family, contains ∼50 different proteins in a mature virion (35). Remarkably, bubblegram imaging, an adaptation of EM, uncovered the presence of an additional, distinctive substructure, called the “inner body,” in the capsids of some jumbo phages infecting *Pseudomonas* species (ФKZ and 201Ф2-1) and Escherichia coli (phages 121Q and phAPEC6) (25, 36–41). The inner body is a spool-like internal proteinaceous structure encased alongside phage genomic DNA. A thorough elucidation of the function of the inner body has yet to be undertaken, but this structure has been thought to arrange genomic DNA in the viral head during genome packaging. Some jumbo phages produce proteins to assemble other unique architectures in the host cytoplasm that benefit phage fitness during the infection cycle. In the following sections, we will elaborate on discoveries, structural features, and biological functions of those phage-derived subcellular apparatuses in a semichronological manner, focusing mostly on *Pseudomonas* jumbo phages.

## JUMBO PHAGES MANUFACTURE PhuZ TUBULINS FOR EFFICIENT PROLIFERATION

### Discovery of tubulin homologs in jumbo phage genomes.

The cytoskeleton is an elaborate network of actin, tubulin, and intermediate filaments (IFs) that determines cell shape and facilitates the transport of intracellular contents in eukaryotic cells (42–44). It had long been thought to be an exclusive feature of eukaryotes. Not until 1991 was FtsZ from Escherichia coli revealed to have a structural role in cell division, and eventually, it was identified as a homolog of tubulin in prokaryotes (45–48). Following that, the actin homolog MreB in Bacillus subtilis and the IF-like protein crescentin in Caulobacter crescentus were identified in 2001 and 2003, respectively (49, 50). Since then, many other prokaryotic cytoskeletal proteins have been documented and demonstrated to be involved in various cellular processes, such as cell division and DNA segregation (51, 52). Nevertheless, the production of cytoskeletal elements by bacteriophages was not anticipated.

In 2012, two tubulin homologs encoded by phages, each of which represented its own independent subgroup of bacteriophage tubulin/FtsZ-like proteins, were first reported (53, 54). The first reported phage-encoded tubulin/FtsZ-like protein (ORF CST189) comes from Clostridium botulinum phage C-st (54). Phage C-st is a *Siphoviridae* member with 185.7-kbp linear dsDNA, which circularizes as a plasmid prophage in the host cell upon infection (55). The C-st tubulin homolog has been designated as belonging to the subgroup TubZ, which is harbored within low-copy-number *Bacillus* plasmids and is responsible for plasmid segregation through interaction with the TubR DNA-binding protein and the centromeric DNA region (*tubS*) containing TubR binding sites (54, 56, 57). Likewise, TubZ_C-st_ functions in the plasmid partitioning for inheritance during the C-st lysogenic life cycle (54). In parallel, by scanning genomic sequence databases, another study found a number of tubulin-like protein sequences encoded in several large phage genomes, ranging from 186 kbp to 316 kbp (53). From there, the second phage-derived homolog of tubulin/FtsZ protein was described and named “PhuZ,” standing for *ph*age t*u*bulin/Fts*Z* (53). PhuZ is the product of *gp59* in the 316.7-kbp genome of Pseudomonas chlororaphis jumbo phage 201Ф2-1 (25). PhuZ was shown to assemble dynamic filaments that position phage DNA at the cell center during the lytic phage cycle, enhancing phage reproduction (53, 58). PhuZ is conserved among numerous jumbo phage genomes, including Pseudomonas aeruginosa jumbo phages ФKZ, ФPA3, KTN4, EL, and SL2, as well as those that infect Erwinia amylovora, *Vibrio*, and *Bacillus* (13, 59, 60). These results suggest that phages with large genomes encode their own cytoskeletal proteins for optimal intracellular reproduction.

### Structure and assembly of PhuZ filaments in *Pseudomonas* jumbo phages.

**FIG 1:**
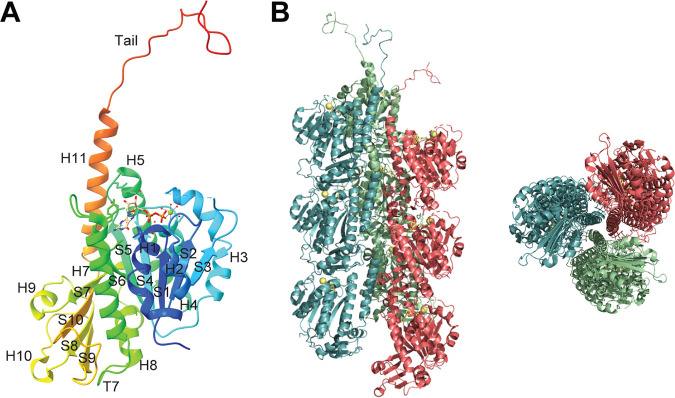
Monomeric and filament crystal structures of phage 201Ф2-1 PhuZ. (A) Cartoon representation of the crystal structure of a PhuZ_201_ monomer annotated with secondary structural elements (PDB ID 3R4V) (53). The bound GDP-Mg^2+^ is shown in ball-and-stick format. (B) Cartoon representation of the crystal structure of PhuZ_201_ filament, with individual protofilaments presented in different colors (PDB ID 3J5V) (67). The bound GDP-Mg^2+^ elements are shown in ball-and-stick format. (Left) Side view; (right) en d-on view.

The crystal structure of PhuZ (Gp59) encoded by P. chlororaphis jumbo phage 201Ф2-1 has been solved at a resolution of 1.67 Å (Fig. 1A) (53). The full-length PhuZ monomer bound to GDP comprises an N-terminal domain containing the nucleotide-binding pocket (GTP-binding domain) connected to a long intermediate domain by the core H7 helix and a small C-terminal GTPase activation domain, similar to the core fold of other tubulins (61, 62). Surprisingly, PhuZ lacks a highly conserved interdomain helix (H6) at its C-terminal region, which plays a key role in the longitudinal interactions within eukaryotic and prokaryotic protofilaments (57, 63–65). Instead, a unique C-terminal tail is formed by a long helix (H11), followed by a short loop, extending out from the PhuZ monomer. Point mutations in the interaction regions or deletion of the knuckle structure at the C terminus of PhuZ completely abolished filament assembly both *in vitro* and *in vivo*, indicating that the C-terminal tail is critical for PhuZ polymerization (53). The distinct C-terminal tail of PhuZ is conserved among many tubulin proteins from *Pseudomonas* jumbo phages, as well as in some clostridial phages, such as C-st (53, 54). In a later study, the crystal structure of a closely related PhuZ protein, PhuZ_Фkz_ (Gp39) from P. aeruginosa jumbo phage ФKZ, was solved; it exhibits significant similarity to PhuZ_201_ (PhuZ from phage 201Ф2-1) (66).

Overexpression of PhuZ with a green fluorescent protein (GFP) label (GFP-PhuZ_201_) revealed dynamic fluorescent filaments extending along the cell length (53, 67). To gain molecular insight into the mechanism of PhuZ filament assembly, the high-resolution 3-dimensional (3D) architecture of a PhuZ filament was determined by cryo-EM, showing that PhuZ assembles a unique three-stranded, right-handed helical filament in which protofilaments twist around each other (Fig. 1B) (67). During polymerization, GTP-bound PhuZ monomers first assemble into dimers through extensive interaction between the C-terminal tail of one monomer and the GTPase domain of an adjacent longitudinal monomer. The longitudinal subunit-subunit interface transits between a relaxed (47 Å) state (53) and a tense state (43.5 Å) (66). Three of these dimers are then laterally organized into a hexameric nucleus, followed by a change in orientation to allow their C termini to face the interior and hold the twisted protofilaments. The hexamer grows by further incorporation of GTP-bound monomers and dimers. Finally, GTP on each subunit in the tense state is hydrolyzed to GDP, and the compacted subunits are not able to return to the extended form. Consequently, energy from the twisting is stored within the helical filament lattice, resulting in the metastable and highly dynamic mature PhuZ filaments. In addition to ФKZ, which shares highly similar structures of PhuZ filaments with those of PhuZ_201_, another *Pseudomonas* jumbo phage, ФPA3, also assembles a three-stranded polymer (60).

### Properties of PhuZ filaments and their intracellular role during phage infection.

By monitoring the entire infection cycle of phage 201Ф2-1 in P. chlororaphis cells *in vivo* at the single-cell level, time-lapse microscopy revealed that PhuZ filaments form after infection and undergo periods of assembly and disassembly until cell lysis (53). PhuZ filaments appear to grow from both cell poles toward the center of the infected cell, suggesting that these filaments polymerize in a polarized manner, similarly to eukaryotic microtubules and some prokaryotic cytoskeletons (68–72). This growth polarity was confirmed utilizing total internal reflection (TIRF) microscopy and rapid time-lapse microscopy to visualize the dynamics of PhuZ filaments *in vitro* and *in vivo*, respectively (58). PhuZ plus (growing) ends are never in equilibrium but rapidly elongate toward the cell center, while minus (assembly-initiating) ends seem to be anchored near cell poles. PhuZ was the first prokaryotic tubulin found to exhibit dynamic instability, a hallmark behavior of eukaryotic microtubules, defined as rapid and stochastic switching between states of filament polymerization and depolymerization (73). Mutations in the conserved catalytic T7 loop that eliminate GTPase activity and prevent GTP hydrolysis result in nondynamic filaments (53, 58). This suggests that as with eukaryotic microtubules, the dynamic instability of PhuZ filaments requires energy released from GTP hydrolysis during polymerization. PhuZ also contains GTP-caps at the tips of prolonged filaments, which stabilize and facilitate the growth of PhuZ filaments (58, 74).

In uninfected cells, PhuZ filaments, though exhibiting dynamic instability, do not exhibit localized spatial organization. However, once the host cell is infected by a phage, PhuZ assembles into a bipolar spindle composed of dynamically unstable filaments with minus ends anchored and stabilized at a single position near each cell pole, indicating that the polar localization of PhuZ relies on phage infection (58). This dependence on phage infection to set up a spindle-like apparatus suggests that a phage-derived factor, yet to be uncovered, is responsible for the organization of spindle assembly. DNA staining of infected cells reveals that after injection at cell poles, the 201Ф2-1 DNA signal increases in size as a result of DNA replication and simultaneously migrates toward the midcell, followed by the formation of a single large structure, initially termed the “infection nucleoid,” in the center of the cell (53, 58). Dual-color DNA fluorescence *in situ* hybridization (FISH) experiments demonstrate that the phage infection nucleoid is composed entirely of phage DNA. Although phage DNA undergoes replication when it travels from the infection site to the cell center, this movement is independent of DNA replication (58). Highly dynamic plus ends of PhuZ filaments frequently interact with and move the infection nucleoid until it is established at midcell. Thereafter, PhuZ filaments continue making frequent contact with the edge of the infection nucleoid until the cell eventually lyses. Expression of a catalytic PhuZ mutant (GFP-PhuZD190A) defective in GTP hydrolysis left the infection nucleoid mispositioned near cell poles rather than at midcell, and the final burst size decreased by 50% (53), suggesting that PhuZ filaments harness dynamic instability to center phage DNA and maximize phage reproduction for optimal fitness.

One of the most important functions of eukaryotic cytoskeletons is to transport intracellular organelles and other structures, such as mitotic chromosomes, throughout the cytoplasm (75). Many viruses exploit microtubule trafficking to travel from the cell periphery to reach an interior cell site for viral replication and to enable newly mature progeny to leave the infected host (76, 77). This cargo trafficking along tubulin filaments was observed for the first time in prokaryotic cells during 201Ф2-1 infection in a 2019 study (59). Live-cell imaging of infected cells in which both capsid protein (Gp200) and PhuZ_201_ were fluorescently labeled showed that empty capsids are assembled on the bacterial cell membrane at ∼45 min after infection and then traffic along filaments of the PhuZ spindle toward the infection nucleus for DNA packaging. Cryo-electron tomography (cryo-ET) of cryo-focused ion beam (FIB) milling demonstrates the attachment of capsids to PhuZ filaments at a higher resolution. Photobleaching studies showed that treadmilling of PhuZ filaments, a behavior described previously for eukaryotic tubulins and some prokaryotic tubulins (56, 78, 79) in which loss of subunits (depolymerization) from the minus end offsets polymerization at the plus end and the filament length remains constant, is responsible for the capsid movement along the PhuZ spindle (58, 59). Importantly, as with dynamic instability, the treadmilling of PhuZ relies on the energy gained from GTP hydrolysis. In addition, after the phage infection nucleoid arrives at the midcell position, the role of PhuZ filaments seems to shift from translocation to rotation of the infection nucleoid (59, 60, 80). There is evidence that infection nucleoid rotation is achieved by treadmilling of PhuZ filaments, which accounts for the uniform distribution of empty capsids that dock on the phage nucleoid to ensure efficient DNA encapsidation (59). Thus, like eukaryotic viruses, jumbo phages have evolved a mechanism for cargo trafficking in the bacterial host by using their own cytoskeletal elements.

In summary, the bacteriophage PhuZ spindle exhibits four critical properties: dynamic instability, the formation of a bipolar array of filaments, spatiotemporal central positioning of phage DNA, and treadmilling. Dynamic instability, driven by nucleotide hydrolysis, results from continuous transitions between filament elongation by the addition of GTP-PhuZ dimers at plus ends and occasional shortening (depolymerization) due to GTP hydrolysis (70, 81). Despite displaying similar intracellular activities, PhuZ_201_ shares <11% sequence identity with eukaryotic αβ-tubulin and only 31% and 46% identity with PhuZ_ФKZ_ and PhuZ_ФPA3_, respectively, suggesting that tubulin sequences can tolerate significant sequence divergence while preserving the ability to assemble filaments with similar dynamic features (60). After the bipolar spindle is assembled, its highly dynamic, unstable three-stranded filament ends position the replicating phage DNA in the cell center by a pushing force. This is reminiscent of the eukaryotic mitotic spindle, which is composed of 13-stranded microtubules consisting of αβ-tubulin subunits, in which cycles of microtubule growth and shrinkage drive replicated chromatids to move toward the metaphase plate at the midcell position (82, 83). Several prokaryotic filaments participate in bacterial plasmid segregation as well, such as a bipolar spindle formed by ParM filaments in Escherichia coli (84, 85) and TubZ polymers from Bacillus thuringiensis (56). The structural and functional similarities among these phage cytoskeletal systems, as well as their complexity compared with bacterial cytoskeletal filaments, suggest that jumbo phage cytoskeletons may have been a source in the evolution of complex eukaryotic cytoskeletal architectures.

Studies on phage tubulins have raised many questions. First, why do bacteriophages require cytoskeletal proteins during host infection? Given that the tubulin homologs have been detected only in phages with large genomes, a large virion might be the main cause of filament formation. Upon expression of phage structural genes during the late stage of infection, intracellular movement of such large structural components as phage capsids and tails to assemble phage progeny at certain locations by diffusion may be restricted in a crowded cytoplasm. The formation of phage tubulins is of great importance for phage subcellular spatial organization and efficient reproduction. A second question is provoked here: why does large phage DNA prefer to be positioned in the cell center? DNA staining of DNase I-treated P. chlororaphis cells after 201Ф2-1 infection has shown that the indigestible phage-encapsidated DNA molecules appear in a rosette-like structure at the edges of the infection nucleoid, resembling a eukaryotic virus factory (53, 59, 86). In conjunction with the spacious advantage of the cell center area, it would be reasonable to hypothesize that the central positioning of phage DNA by tubulin may be advantageous for *in vivo* phage development. Third, all *in vivo* experiments to date have been performed by expressing PhuZ proteins from a plasmid, and it has been pointed out that a functional PhuZ spindle is assembled only when PhuZ proteins meet a threshold concentration in the cell. It would be of interest to explore the natural cellular levels of PhuZ during viral infection. Last, by utilizing advanced technologies such as superresolution microscopy, many other questions could be addressed in the future, such as whether the PhuZ spindle is associated with host factors to facilitate phage postinfection development, how individual PhuZ filaments interact with the infection nucleus, and whether PhuZ filaments participate in other cellular processes. Understanding these questions would shed light on the evolutionary relationships between the cytoskeletal proteins of viruses, prokaryotes, and eukaryotes.

## JUMBO PHAGES ASSEMBLE A NUCLEUS-LIKE STRUCTURE DURING VIRAL LIFE CYCLES

### Discovery of a proteinaceous shell encoded by jumbo phages.

Many eukaryotic viruses reorganize cellular components after infection and build specific subcellular microenvironments known as “virus factories” or “viroplasms,” where viral genome replication and morphogenesis take place (86, 87). Those viral macrostructures allow eukaryotic viruses to concentrate viral components and recruit host cell organelles, such as mitochondria and ribosomes, at certain locations in the cytosol, in order to facilitate viral metabolic reactions and achieve efficient progeny reproduction (88, 89). The biogenesis of virus factories involves the rearrangement of cell membranes and cytoskeleton (87, 88). Virus factories also provide physical protection for viral genomes against cellular antiviral defenses (86, 90). However, no such structures have been reported for bacteriophages as of 2017.

**FIG 2:**
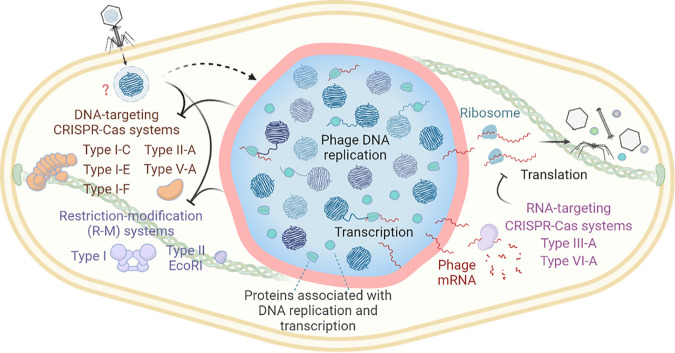
The bacteriophage nucleus-like shell combats antiphage defense mechanisms. Jumbo phages assemble a proteinaceous shell that separates phage genomes from the bacterial cytoplasm and segregates proteins according to function during viral replication. DNA replication and transcription occur inside the shell, while translation and metabolic processes take place in the cytoplasm. The shell physically shields phage genomes from attack by DNA-targeting CRISPR-Cas systems and restriction enzymes. However, RNA-targeting CRISPR-Cas systems can cleave phage transcripts in the cytoplasm. It remains unknown how phage DNA is protected upon injection (depicted as a dashed curve and a question mark).

In the investigation of the role of other phage proteins in PhuZ filament formation, Gp105 of phage 201Ф2-1 was identified as the first and most highly expressed protein after infection, based on protein mass spectrometry (80). Surprisingly, fluorescence microscopy showed that GFP-Gp105 assembles a nucleus-like compartment to enclose phage DNA at the cell center, and cryo-ET confirmed the existence of such a structure during infection. This proteinaceous shell serves as a barrier and has the ability to segregate proteins according to function (Fig. 2). Proteins involved in DNA replication and transcription localize inside the shell, whereas proteins involved in translation, such as ribosomes, and nucleotide metabolic processes localize outside the shell in the cytoplasm. This compartmentalization of proteins suggests that the Gp105 shell separates cellular activities for phage development, with genome replication and transcription taking place inside the shell, while translation and metabolism occur in the cytoplasmic space. In this manner, this structure is very similar to that of the eukaryotic nucleus, which generates separate compartments for transcription and translation.

The presence of the shell structure suggests that PhuZ polymers interact with shell proteins, rather than with replicating phage DNA, for spatial movement of the phage nucleoid. After being centered at midcell, the shell oscillates and rotates along the central axis as a result of the constant treadmilling and pushing force imposed by PhuZ filaments (58, 80). Likewise, phage empty capsids migrate along filaments of the PhuZ spindle to the shell (formerly “infection nucleoid”), the surface of which serves as a platform for capsids to dock and package phage DNA. Two other *Pseudomonas* jumbo phages, ФKZ and ФPA3, also encode homologs of Gp105 shell protein, Gp054 and Gp053, respectively, to form a similar proteinaceous shell surrounding phage DNA that segregates phage proteins based on their functions (60). All three phages set up a bipolar spindle that exploits the dynamic instability of their PhuZ filament ends to position the nuclear compartment at midcell. Most recently, a *Serratia* jumbo phage, PCH45, was also shown to produce a nucleus-like structure and to encode a PhuZ protein (Gp187) and a shell protein (Gp202) (91). Despite having low sequence identity with those of *Pseudomonas* phages, PCH45 shell protein can assemble into a nucleus-like compartment to enclose phage DNA upon infection as well. This suggests that the phage nucleus and spindle structures, as well as their biological properties and cellular functions, are characteristics conserved among some jumbo phages.

Much less is known about how phage molecules such as proteins and mRNA are selectively transported between the compartment interior and the cytoplasmic space. The nucleus of a eukaryotic cell exploits a selective transport system to transfer macromolecules between the nucleus and the cytosol (92). Nuclear proteins rely on active nuclear localization signals (NLSs) that are recognized by nuclear import receptors for import into the nucleus. Conversely, large molecules such as RNA and ribosomal subunits require their own nuclear export signals (NESs), as well as nuclear export receptors, to depart the nucleus (93). Whether or not those phage molecules also contain sorting signals in their sequences awaits further characterization.

A recent study has provided hints about the selective transportation of the shell. In this study, fusion of a restriction enzyme, EcoRI, with a shell-internalized recombinase from ФKZ relocates EcoRI into the shell, whereas the same does not work for Cas9, which seems to get stuck on the shell surface, perhaps due to its size, 158 kDa (1,368 amino acids), which is much larger than that of EcoRI, 31.5 kDa (278 amino acids) (94). Accordingly, we speculate that while the size of macromolecules may be a driver, it is not the only deterministic factor in the selective transportation mechanism of the phage shell, since the even smaller EcoRI (unfused to the internalized protein) is excluded. In addition, it remains unclear whether the shell is perforated by any pore-like structures to allow molecules to cross, resembling the nuclear pore complexes on the eukaryotic nuclear envelope (95). In fact, as mentioned above, phage capsids relocate from the plasma membrane, where they are assembled, to the phage nuclear compartment. Thereafter, phage genomes inside the shell are delivered into the empty capsids that remain on the surface of the nucleus shell to form mature capsids in the cytoplasm. Phage mRNA also needs to be translocated, after being transcribed within the shell, to the cytoplasm for translation. It is evident that there must be mechanisms for nucleic acids and proteins to pass through dedicated channels or pores generated via local rearrangements in the shell wall.

### The shell protects jumbo phages against broad DNA-targeting immune systems.

It is well known that phages have evolved various strategies to circumvent bacterial antiviral machineries that target phage nucleic acids, such as CRISPR-Cas (clustered regularly interspaced short palindromic repeats [CRISPR]–CRISPR-associated proteins) and restriction-modification (R-M) systems, thereby creating an everlasting evolutionary battle between bacteria and phages (96, 97). Those strategies include modifications of the phage genome to avoid being targeted and expression of anti-CRISPR (Acr) proteins or anti-RM proteins such as Ocr and DarA/DarB to directly inhibit host immune activities (96, 98–100). Early in 2020, two independent studies discovered that three jumbo phages, ФKZ, ФPA3, and PCH45, are broadly resistant to endogenous CRISPR-Cas systems from *Pseudomonas* and *Serratia*, such as subtypes I-C, I-E, and I-F, and also to exogenous CRISPR-Cas systems, such as subtype II-A from Streptococcus pyogenes and V-A from Moraxella bovoculi (91, 94). In addition, ФKZ is resistant to two restriction endonucleases, HsdR and EcoRI, from type I and II R-M systems, respectively (94). However, no homologs of any known *acr* genes or DNA modification genes can be detected in the genomes of either ФKZ, ΦPA3, or PCH45, indicating that these three jumbo phages have developed a distinct, previously unsuspected immunity evasion strategy. Nonetheless, ФKZ and PCH45 are susceptible to RNA-targeting CRISPR-Cas systems, the type VI-A system adapted from Listeria seeligeri, and the type III-A system naturally present in *Serratia*, suggesting that bacterial immunity can still be achieved by targeting phage mRNA.

The broad resistance to bacterial immune nucleases is attributed to the proteinaceous nucleus-like compartment that is assembled by all three phages during the viral infection cycle (Fig. 2). Fluorescence microscopy provides direct evidence that the labeled Cas proteins and restriction endonucleases are excluded from the compartment and that the shell behaves like a physical protective barrier against DNA-targeting enzymes, preventing their access to phage genomes that are enclosed in the compartment (91, 94). When the EcoRI restriction enzyme is fused to the ФKZ recombinase (ORF152), the fused EcoRI is internalized and is able to cleave the ФKZ genome within the shell, thereby protecting host cells (94). Scanning of thousands of bacterial genomes shows that spacers that match the genome sequences of those shell-forming jumbo phages for type I CRISPR-Cas systems are not found (94) but that such spacers can be detected in CRISPR arrays of type III systems (91). This indicates that this group of jumbo phages generally resists DNA-targeting type I CRISPR-Cas systems but is sensitive to RNA-targeting type III immunity in nature.

The physically protective function of the shell against bacterial DNA-targeting immunity is observed in two evolutionarily distant jumbo phages, suggesting that this novel viral antidefense mechanism may be widespread among jumbo phages. On the other hand, the shell is futile in the presence of RNA-attacking immunity, shedding light on the importance of RNA-targeting immune pathways from an evolutionary perspective. The emergence of bacterial RNA-targeting CRISPR-Cas systems may be not only a response to combat RNA phages but also a countermeasure against phage DNA protective mechanisms such as base modifications in phage genomes and genome segregation mechanisms. Such a proteinaceous compartment might not only confer a selective advantage on large phages by acting as a powerful means of thwarting any damage to the phage genetic material but might also be hypothesized to competitively exclude other phages, such as the resident prophage, by sequestering cellular resources to ensure their own infection success (101). However, the production of such a proteinaceous compartment might impose a potential fitness cost, thereby leading to a much smaller burst size (16 for phage 201Ф2-1 [53] and 6 to 8 for phage KTN4 [102]) than those of phages with smaller genomes (∼100 for phage λ [103] and ∼200 for phage T4 [104]).

### Current picture of assembly and central positioning of a phage nucleus by PhuZ.

**FIG 3:**
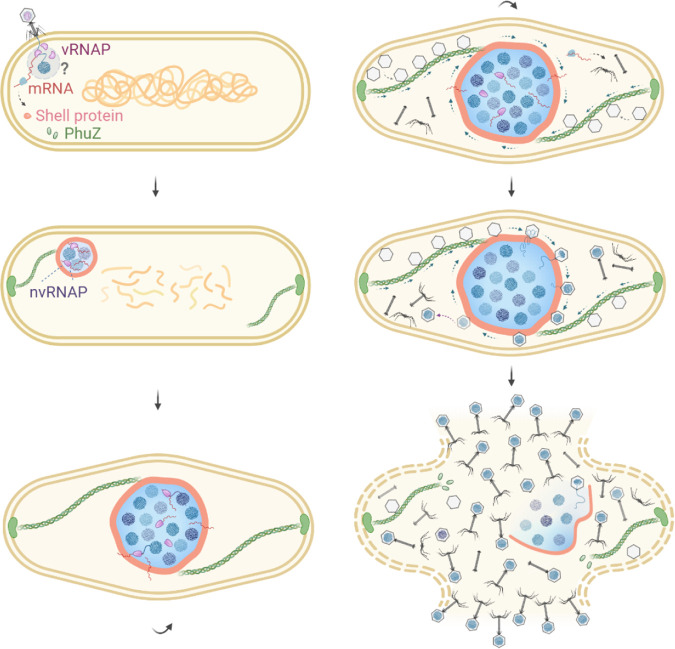
Intracellular development of jumbo phages after infection of a bacterial host. Upon encountering a bacterial host, the phage injects its genomic DNA, accompanied by the translocation of virion RNA polymerases (vRNAPs) that are present inside the viral capsid. vRNAPs transcribe early phage genes, including genes encoding the shell protein and PhuZ. While the host chromosome is degraded, PhuZ forms filaments anchored at each cell pole and gradually establishes a bipolar spindle in the cell. Meanwhile, the shell proteins assemble a nucleus-like compartment to sequester phage DNA from the cytoplasm and protect phage DNA against host defense systems. Nonvirion RNAPs (nvRNAPs) appear later inside the compartment and are responsible for expression of the late phage genes, including viral structural genes. As phage DNA replicates inside the shell, the compartment grows and is pushed toward the cell center by dynamically unstable filaments of the PhuZ spindle. The cell becomes elongated and forms a bulge at midcell. After the phage nucleus is settled in the cell center, treadmilling PhuZ filaments rotate the shell locally and deliver empty phage capsids from the cell inner membrane to the phage nucleus for subsequent DNA encapsidation. Rotation of the phage nucleus facilitates even distribution of phage capsids around the shell surface, ensuring efficient DNA packaging. Eventually, filled capsids are released from the shell and assemble into mature phage particles together with other viral structural components that are generated in the cytoplasm, followed by cell lysis to release phage progeny to the environment.

The discovery of the shell advances our understanding of how PhuZ filaments position the phage nucleoid and participate in phage progeny packaging over the viral life cycle. A combination of time-lapse fluorescence microscopy and cryo-electron tomography presents a fascinating picture of the spatiotemporal postinfection development of these jumbo phages (Fig. 3). Upon infection, highly expressed shell proteins appear as a small compartment, and PhuZ rapidly assembles filaments to build a three-stranded, bipolar spindle. The shell sequesters phage DNA from the host cytoplasm, allowing viral DNA replication and transcription to occur inside the shell. Over time, the compartment gradually grows in size and is pushed by PhuZ filaments toward the cell center. After the shell arrives at the midcell area, opposing filaments of the PhuZ spindle keep oscillating and rotating the shell, allowing newly synthesized phage capsids, which are directionally delivered by filament treadmilling, to be evenly distributed on the shell surface. Eventually, after being filled with phage genomes, mature capsids are released from the nucleus and then couple with tails present in the cytoplasm to assemble mature progeny particles, followed by cell lysis. Throughout the entire viral life cycle, the nucleus-like compartment serves as a proteinaceous barrier to segregate bacterial host and phage proteins based on their cellular functions; more importantly, it provides a protective mechanism to prevent phage DNA from being destroyed by host antiviral defense systems.

## PHAGE THERAPY WITH JUMBO PHAGES

With the incredible power to effectively kill bacteria, bacteriophages have a long history of being used for the control and clearance of antibiotic-resistant superbugs and other problematic bacteria in clinical trials and environmental systems (105–107). Phages offer many advantages over conventional antimicrobial agents, such as efficient self-propagation in the presence of bacteria, high specificity for target pathogens, fast and inexpensive isolation, limited collateral damage due to host specificity, and reduced potential environmental impact (105, 108, 109). With substantial potential in the treatment of human bacterial diseases as well as in agriculture, veterinary science, industry, and food safety, phage therapy has been considered a realistic alternative to antibiotic treatments in the age of multidrug resistance (106, 110). Although phage therapy holds great promise for biocontrol, it still faces many challenges, one of which is contributed by bacteria, which possess antiviral defenses to abolish phage infection (111). Consequently, jumbo phages with the ability to form a genome protective shell that confers broad resistance to DNA-targeting bacterial immunity during infection have become suitable candidates to overcome this challenge. Moreover, in addition to being virulent phages that commit to the lytic life cycle, jumbo phages have been reported to harbor extra genes responsible for host lysis, genomic DNA replication and transcription, and nucleotide metabolism, leading to reduced dependence on the host strain and a host range wider than those of phages with smaller genomes (10). These features could make jumbo phages ideal for phage therapy applications, although resistance mechanisms are still expected.

While a variety of studies have recently investigated the plausibility of the therapeutic use of various jumbo phages for microbial control (14, 29, 112, 113), many limitations remain. First, jumbo phages have not received comprehensive characterization, and whether those hypothetical genes encoding viral proteins with unclear functions have any side effects or even are lethal to the treated subjects is not predictable yet. Second, some jumbo phages, such as the ФKZ-related phage AR9 (26), exhibit frequent horizontal gene transfer (HGT) and are capable of genetic exchange between various bacterial hosts. This gene transfer could accelerate the acquisition of resistance by pathogenic bacteria against therapeutic phages, leaving phage therapy ineffective (114). Third, the loss of a single cell surface receptor can confer resistance to phage infection, as with many other phages (97, 115). Fourth, the broad host spectrum might increase the possibility of off-target killing. Therefore, the usage of jumbo phages needs to reach a compromise between host specificity and universality for optimal treatment. Finally, genetic manipulation of jumbo phages with such large genomes and an obligatory lytic life cycle is currently challenging. Genetic modification of jumbo phages is expected to alter their natural properties and enhance their efficiency and biosafety in killing bacteria. In sum, future investigation of jumbo phages will undoubtedly uncover more fascinating features that benefit basic phage biology, enrich evolutionary perspectives, and enhance the therapeutic use of phage in diversified applications.

## EVOLUTIONARY CONSIDERATIONS

Viruses are ubiquitous intracellular parasites that rely to a great extent on host materials and metabolic systems for their reproduction. Although eukaryotic viruses and bacteriophages infect different domains of life and differ dramatically in virion morphology and genetic composition, subsets from each group are structurally and evolutionarily related (116–118). The herpesviruses infecting eukaryotes and the *Caudovirales* order of phages share similarities in the structures of their capsid proteins, as well as in capsid assembly and DNA-packaging strategies (117, 119). The recently discovered nucleus-like structure derived from jumbo phages is reminiscent of viral factories that are built by most nucleocytoplasmic large DNA viruses (NCLDV) of eukaryotes, such as vaccinia virus and mimivirus (120, 121). Not only are viral factories able to concentrate replicase proteins, viral genomes, and host proteins required for viral replication, but they also function to protect against cellular antiviral defense systems, as in the case of the jumbo phage nucleus (90). It has been hypothesized that the eukaryotic nucleus might have originated from the viral factory produced by an ancient member of NCLDV (122). Since prokaryotes lack a membrane-bounded subcellular compartment to surround their chromosomal DNA, we may speculate that eukaryotic viruses and nucleus-producing large bacteriophages arose from a relatively small number of primordial ancestries. Indeed, comparative genomic and proteomic analyses have identified evolutionary connections between bacteriophages and giant eukaryotic dsDNA viruses, as well as large DNA transposons and linear DNA plasmids (118, 119, 123). In particular, an evolutionary scenario suggests that the NCLDV may have evolved from a group of bacteriophages as a result of incorporation of numerous eukaryotic and bacterial genes, with concomitant loss of most of the phage genes except for core genes essential for viral genome replication and virion formation (124). Since the majority of viral genes from jumbo phages encode phage proteins with unknown functions, functional characterization of these genes may shed light on the evolutionary transition from cell-dependent organisms, such as phages and large viruses, to cell-independent prokaryotes and complex eukaryotes.

## PERSPECTIVES

It is compelling that some large bacteriophages have evolved such vigorous avenues to proficiently localize and coordinate their activities within the crowded bacterial cytoplasm. Remarkably, the versatile compartment built by these phages equips viral genomes with a “safe room,” excluding a variety of bacterial DNA-targeting immune enzymes. The effectiveness of RNA-targeting enzymes demonstrates the endless arms race between bacteria and phages under natural conditions as one develops strategies to outmaneuver the other. All these discoveries remind us of how much we still have left to investigate with regard to the group of jumbo phages. We anticipate that in the coming years, exploration of interactions between jumbo phages and their prokaryotic predators will uncover more surprising phenomena, which, in turn, will drive immunological innovation and revolutionary phage therapeutics.
